# Relevance of Hydrogen Bonds for the Histamine H2 Receptor-Ligand Interactions: A Lesson from Deuteration†

**DOI:** 10.3390/biom10020196

**Published:** 2020-01-29

**Authors:** Mojca Kržan, Jan Keuschler, Janez Mavri, Robert Vianello

**Affiliations:** 1 Institute of Pharmacology and Experimental Toxicology, Faculty of Medicine, University of Ljubljana, Korytkova 2, SI–1104 Ljubljana, Slovenia; mojca.limpel@mf.uni-lj.si (M.K.); jan.keuschler@gmail.com (J.K.); 2 Laboratory for Computational Biochemistry and Drug Design, National Institute of Chemistry, Hajdrihova 19, SI–1001 Ljubljana, Slovenia; janez.mavri@ki.si; 3 Division of Organic Chemistry and Biochemistry, Ruđer Bošković Institute, Bijenička cesta 54, HR–10000 Zagreb, Croatia

**Keywords:** computational chemistry, DFT calculations, deuteration, heavy drugs, histamine receptor, receptor activation, hydrogen bonding

## Abstract

We used a combination of density functional theory (DFT) calculations and the implicit quantization of the acidic N–H and O–H bonds to assess the effect of deuteration on the binding of agonists (2-methylhistamine and 4-methylhistamine) and antagonists (cimetidine and famotidine) to the histamine H2 receptor. The results show that deuteration significantly increases the affinity for 4-methylhistamine and reduces it for 2-methylhistamine, while leaving it unchanged for both antagonists, which is found in excellent agreement with experiments. The revealed trends are interpreted in the light of the altered strength of the hydrogen bonding upon deuteration, known as the Ubbelohde effect, which affects ligand interactions with both active sites residues and solvent molecules preceding the binding, thus providing strong evidence for the relevance of hydrogen bonding for this process. In addition, computations further underline an important role of the Tyr250 residue for the binding. The obtained insight is relevant for the therapy in the context of (per)deuterated drugs that are expected to enter therapeutic practice in the near future, while this approach may contribute towards understanding receptor activation and its discrimination between agonists and antagonists.

## 1. Introduction

With at least 800 unique members, human G protein-coupled receptors (GPCRs) encompass the largest superfamily of cell-surface receptors, which translate external cell signals into measurable stimuli resulting in precise cell responses [1,2]. Some examples of physiological and pathological responses controlled by GPCRs include the neurotransmission, secretion, contraction, cell growth and differentiation, which make them excellent specific targets for a variety of therapeutic approaches. Some estimates predict that GPCRs embody around 30% of the existing drug targets, while their therapeutic potential might be even larger [3,4].

Receptor ligands are described as (i) agonists if they are capable of activating the receptor and display full efficacy, (ii) partial agonists showing only partial biological response after receptor activation, (iii) antagonists if their binding to receptor does not involve any change of basal receptor activity, or (iv) inverse agonist, as ligands with the negative efficacy. Considering thermodynamic aspects, the binding of antagonists to their targets is typically associated with more favorable interaction free energies (affinities) than it is with agonists. The available GPCR crystal structures broadly classify three discrete conformations: (1) an “inactive state” when the receptor is crystallized in a complex with an antagonist or inverse agonist, (2) an “agonist-bound state” lacking the G protein, and (3) a “fully-active state” resulting from a ternary complex involving the receptor, an agonist and the G protein (or G protein surrogate), where all these states are linked by intermediate conformations. The mechanisms that control GPCR ligand binding and receptor activation are remarkably complex and have been, until quite recently, hindered by a lack of structural knowledge of active and inactive states. The design of new therapies with a required activating or inactivating profile could be significantly improved with a more complete knowledge of how GPCRs operate at a molecular level, so that this information could then be transferred to the ligand in question.

Literature reports many studies on how GPCRs are activated and transmit their signals from the extracellular site through to the G protein coupling domain on the intracellular side [5]. Alternatively, we have been interested in how different ligands, agonists and antagonists, bind to the receptor binding site, and whether these processes are modulated upon deuteration, which could potentially reveal factors affecting receptor´s distinction between its agonists and antagonists. This idea offers an interesting extension of the recently observed fact that scent recognition is affected by deuteration, namely that fruit flies (*Drosophila melanogaster*) can discriminate between several isotopomers that have hydrogens replaced with deuteriums [6]. In addition, there is an remarkable observation that fruit flies, trained to distinguish deuterated olefins, also differentiate non-deuterated olfactants with strong infrared (IR) peaks in the 2300 cm^−1^ range [6,7], suggesting that a difference between C–H and C–D vibrational modes is a prominent feature of odorant perception in this species. A likely rationalization of this phenomena is that there is a spectroscopic component to olfaction [7,8,9], or that this is due to inelastic scattering effects [10]. Isotopic substitution of a hydrogen (H) with heavier deuterium (D) shifts the C–H stretching frequency from the 2850–3100 cm^−1^ range into the 2300 cm^−1^ range. Still, only few molecules absorb in this IR region and there is little or no biological need or evolutionary pressure that we know for detecting deuterated compounds. Unsurprisingly, this theory faced extensive skepticism, since it contradicts a more commonly proposed model in which both the geometric shape and chemical nature of the olfactant are the primary components of olfactory reception [11], being supported by the fact that two chiral molecules may smell very differently. It is worth emphasizing here that in the receptor activation processes, like in the enzyme catalysis, dynamical effects are likely irrelevant [12].

In our previous work [13], we used a combination of experimental and computational techniques to investigate the effects of deuteration on the binding of histamine to the ^3^H-tiotidine-labeled histamine H2 receptor in neonatal rat astrocytes. The affinity of histamine to displace bound tritiated tiotidine was significantly higher in deuterated (namely by 0.73 kcal mol^–1^) than in non-deuterated environment, thus confirming the relevance of hydrogen bonding in the process of agonist-receptor binding. Density functional theory (DFT) calculations on the cluster system, extracted from the homology H2 model, along with the implicit quantization of the acidic N–H and O–H bonds demonstrated that these changes in the binding can be rationalized by the altered strength of the hydrogen bonding upon deuteration known as the Ubbelohde effect [14], while reproduced the measured affinity difference with excellent agreement at 0.51 kcal mol^–1^. The clinical relevance of the ligand H/D substitution lies in the context of perdeuterated and thus more stable drugs that are expected to enter therapeutic practice in the near future. Selective incorporation of deuterium in place of hydrogen has the unique effect of retaining the biochemical potency and selectivity of physiologically active compounds while, in select instances, enabling substantial benefits to the overall pharmacological profile of the resulting compounds [15,16], including extension of elimination half-life, optimization of dose and dosing regimen, and mitigation of risks associated with drug-drug interactions [17]. This strategy has attracted significant commercial interest and has been the subject of recent reviews [18,19,20]. When applied to compounds with well-understood therapeutic utility, selective deuteration can be a unique risk-reduced approach to creating new chemical entity drugs that address significant unmet medical needs. With more deuterium-containing compounds entering clinical evaluation, it appears increasingly likely that the approach will succeed in producing important new remedies [21,22].

**Figure 1:**
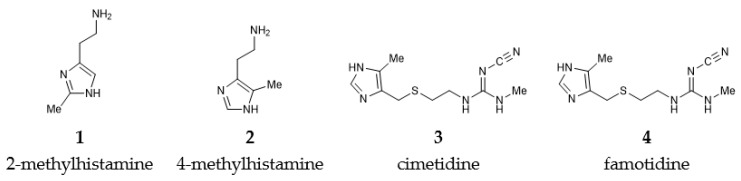
Schematic representation of the studied histamine H2 receptor agonists and antagonists.

The present work builds on our earlier results [13] and considers here two histamine H2 receptor agonists, 2-methylhistamine and 4-methylhistamine, and two antagonists, cimetidine and famotidine (Figure 1), and computationally investigates the effect of deuteration on their affinity using a larger cluster model of the receptor binding site. The presented analysis is likely to contribute towards understanding receptor activation, while the in silico discrimination between agonists and antagonists, based on the receptor structure, remains a distant goal.

## 2. Computational Details

The starting point of our analysis was the homology model of the histamine H2 receptor built earlier [13], while the structure with the bound histamine was used as a template to manually bind agonists 2-methylhistamine (**1**) and 4-methylhistamine (**2**), and antagonists cimetidine (**3**) and famotidine (**4**) into the cluster model of the binding site. In doing so, we tried several conformations for each ligand to avoid errors associated with arbitrary spatial arrangements and proceeded with the most stable complexes. The binding site was composed of Asp98, Asp186 and Tyr250 residues, in analogy with our previous study [13], enriched here with Lys175 and Thr190. According to the PROPKA 3.1 analysis [23] carried out on the entire homology structure [13], these residues were considered as deprotonated anions (Asp98, Asp186), protonated cations (Lys175), and unionized systems (Tyr250 and Thr190). Moreover, **1** and **2** were modeled as monocationic species in their most stable π–tautomeric forms in both the aqueous solution and receptor binding site, as suggested in the literature [24], while cimetidine **3** (p*K*_a,EXP_ = 6.80 – 6.93) [25] and famotidine **4** (p*K*_a,EXP_ = 6.76 – 6.89) [26] were considered as neutral systems in all phases. The latter p*K*_a_ values indicate that, at physiological pH = 7.4, there will be a notable population of monoprotonated **3** and **4**, yet these will be outnumbered by the unionized analogues, which will mainly be responsible for the receptor binding. The mentioned residues were truncated at their α-carbon atoms, which were kept fixed in all calculations during the geometry optimization with the DFT M06–2X methodology in conjunction with the 6–31+G(d,p) basis set, using the Gaussian16 software [27]. Total molecular electronic energies were extracted without thermal corrections, thus the results reported here correspond to differences in electronic energies. The effect of the rest of the protein environment was considered with the CPCM implicit solvation model using a dielectric constant of ε = 4.0, as suggested by Himo and co-workers [28], and a dielectric constant of ε = 78.4 for the aqueous solution, all in line with our previous reports [13], where we also demonstrated that a potential increase in the former dielectric constant to ε = 20.0 lowers the accuracy of the obtained results and even predicts wrong trends among ligands. Additionally, in our experience, such a truncated cluster-continuum model of the entire protein turned very useful in rationalizing various aspects of the catalytic activity [29], selectivity [30] and inhibition [31] of the monoamine oxidase family of enzymes, and is broadly used by different groups to decipher various biological phenomena [32,33,34,35,36], which justifies its use here. We note in passing that, despite its practical usefulness, the proposed value of ε = 4 is, in some cases, apparently too small to prevent the proton transfer from protonated to anionic residues, which then occurred spontaneously during the geometry optimization, provided the involved pair is in close vicinity. Still, this is consistent with literature reports on the prevalence of neutral over ionic hydrogen bonds in low polarity environments such as can be the case with some protein interiors [37,38,39,40].

Literature reports on a range of methods for the quantization of nuclear motion; yet, these are limited to only a few degrees of freedom. However, here we have several critical protons directly involved in the H2 receptor-ligand recognition and water hydration process. As such, we decided to employ an approximate empirical treatment of the nuclear quantum effects involved in the binding. To evaluate the effects linked with the isotopic substitution, we considered the work by Bordalo and co-workers [41], who used a very precise neutron diffraction analysis on alanine zwitterion to show that deuteration reduces the electrostatic attraction in the acidic N–D bonds by 2.3% relative to the matching N–H bonds. This results in the shortening of the N–D distances, as already noticed in various papers [42,43,44,45,46]. Considering both of these aspects, we imposed the empirical quantization in the following way. Initially, all systems were fully optimized, corresponding to the case with lighter H nuclei. After that, all acidic N–H and O–H bonds were shortened by 2.3% and kept frozen during the optimization of other geometric parameters, thus mirroring the situation with heavier D nuclei. The choice of such a computational setup was facilitated by its success in reproducing changes in the binding of histamine to the histamine H2 receptor binding site induced by deuteration [13].

## 3. Results and Discussion

In order to evaluate differences in the binding of investigated ligands to the H2 receptor upon deuteration, we must recall that this process occurs in two stages. Initially, a ligand is located in the aqueous solution surrounded by water molecules, while, ultimately, it is found within the receptor binding site. As such, the thermodynamic picture of the entire process involves two components: the energy of hydration, Δ*E*_HYDR_, and the energy of interaction with the receptor binding site, ΔΔ*E*_INTER_, in the same order. With this in mind, the deuteration-induced change in the overall binding energy, ΔΔ*E*_BIND_, becomes a difference in the mentioned hydration and interaction energies (Table 1):

$$\Delta \Delta E_{BIND}\left(H\to D\right)=\Delta E_{HYDR}\left(H\to D\right)-\Delta E_{INTER}\left(H\to D\right)$$

**Figure 2:**
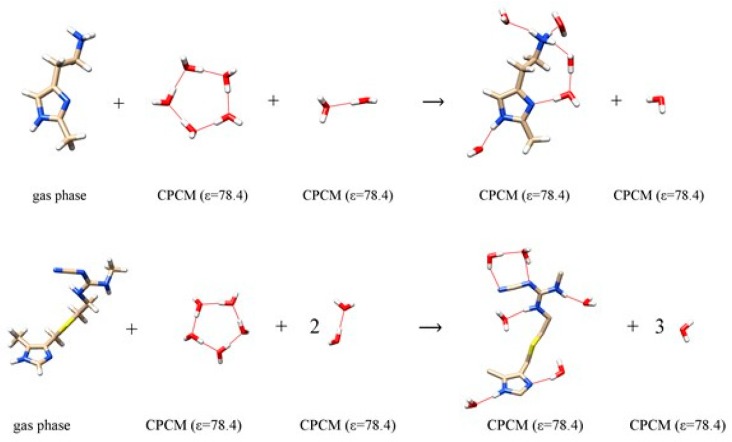
Computational scheme of 2-methylhistamine monocation **1** (top) and cimetidine **3** (bottom) interacting with water molecules to calculate the hydration energy. The selection of the dielectric constant is specified in round brackets. Analogous schemes were employed to calculate the hydration energies for 4-methylhistamine **2** and famotidine **4** and are presented in Figure S1.

In other words, the substitution of exchangeable H-atoms with deuterium results in changes in the corresponding geometric parameters, but also in the altered energies of the matching hydrogen bonds. Disruption of this delicate and fine-tuned equilibrium has potential effects on the ligand-receptor binding affinities, which could be harnessed for therapeutic uses. As a note, deuteration typically attenuates hydrogen bonding interactions, yet, since any deuteration-prompted changes in the binding affinity are brought about as a difference between two quantities, the overall effect can be either positive or negative, depending on the ligand. Previously, we demonstrated that if one uses a cluster-continuum methodology to investigate aqueous phase phenomena, it requires at least five explicit solvent molecules to accurately model the conformational and tautomeric properties of the physiological histamine monocation in water [47]. In this context, we constructed a set of reactions (Figure 2) to evaluate the hydration energies, Δ*E*_HYDR_, of each ligand in water. This approach maintains the number of hydrogen bonds on each side of the equation, being fully in line with the model of homodesmotic reactions [48]. For the histamine monocation, it gives the water hydration free energy of –67.5 kcal mol^–1^ [47], being in a very close quantitative agreement with the MP2/6–31++G(2d,2p) and Langevin dipole calculated value of –68.6 kcal mol^–1^ [49], thus lending credence to the choice of this computational setup.

**Figure 3:**
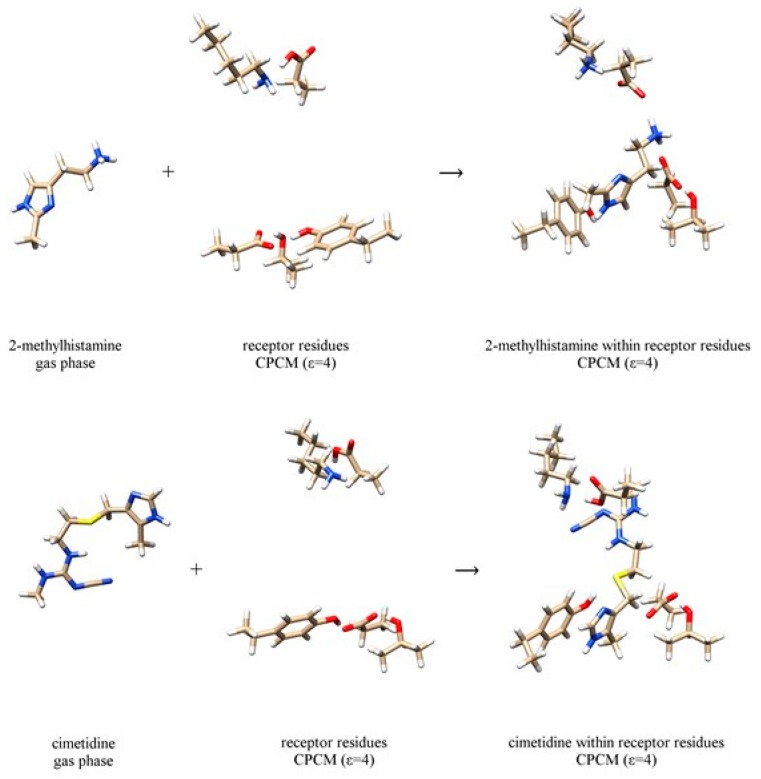
Computational scheme to calculate the interaction energy between 2-methylhistamine monocation **1** (top) and cimetidine **3** (bottom) with the receptor binding site. The selection of the dielectric constant is specified in round brackets. Analogous schemes for 4-methylhistamine **2** and famotidine **4**, and are presented in Figure S2.

On the other hand, the interaction energies, Δ*E*_INTER_, are obtained utilizing a scheme described in Figure 3, which considers placing a ligand from the gas-phase into the cluster model of the binding site. It turns out that all four ligands **1**–**4** are stabilized in water as evidenced in negative hydration energies, Δ*E*_HYDR_. These are significantly higher for **1** and **2** (around –67 kcal mol^–1^) than for **3** and **4** (between –14 and –20 kcal mol^–1^), which is not surprising given that the first two systems are charged monocations protonated at the corresponding free ethylamino groups. A slightly higher hydration free energy of **3** over **4** can be qualitatively related with its around 110 times higher solubility in water [50], which lends credence to these results. In addition, Δ*E*_HYDR_ is somewhat higher for **2** than for **1**, which appears to be in line with slightly higher basicity of the former, as seen in p*K*_a_(**2**) = 7.3 and p*K*_a_(**1**) = 7.1 [24]. In D_2_O, Δ*E*_HYDR_ values are a bit lower, except for **2**, where it is 0.17 kcal mol^–1^ higher. This implies that, in all systems but **2**, the hydration works in the direction of promoting the binding of deuterated systems to the receptor.

In contrast, interaction energies, Δ*E*_INTER_, are consistently much higher than Δ*E*_HYDR_ values due to the polar nature of the binding site involving charged amino acid residues. It is worth pointing out that agonists **1**–**2** show similar receptor binding motif, already observed for histamine [13], involving Asp98, Asp186 and Tyr250 residues, which predominantly bind **1** and **2** to their ethylamino group, N–H moiety on the imidazole ring and imino nitrogen atom of the same group, respectively (Figure 3). It is very important to underline that this binding pattern is different from the model by Birdsall and co-workers [51] proposed on the basis of the site-directed mutagenesis studies carried out by Gantz and co-workers [52], which suggested Thr190 to bind histamine on its imino nitrogen. However, as we have already noticed [13], Thr190 residue is not appropriately positioned for such interaction. Instead, it is located in the close vicinity of the Asp186 residue forming hydrogen bonding interactions to it. **3** and **4** are bulkier and unionized molecules, and their interaction energies Δ*E*_INTER_ are lower to those of **1** and **2**. For **3** and **4**, these cluster between –27 and –32 kcal mol^–1^ in both solvents, while for the other two systems one notices a large increase in Δ*E*_INTER_ value for **2** relative to **1**. Figure 3 reveals that this is due to the unfavorable steric hindrance of the imidazole methyl group in **1** that is positioned close to the Tyr250 residue, thus favoring π·····π and C–H·····π interactions instead of Tyr250–OH·····N(imidazole) hydrogen bonding. In **2**, the analogous methyl group in the 4-position points in the other direction, thus allowing Tyr250 to optimize its interaction with the ligand.

Combining the mentioned hydration and interaction energies, one arrives to the overall binding energies for the receptor-ligand recognition, which yield interesting conclusions. It turns out that, for antagonists cimetidine **3** and famotidine **4**, deuteration produces almost identical binding energies Δ*E*_BIND_. More precisely, both systems slightly increase their interaction with the receptor upon deuteration, but only marginally, by –0.09 and –0.05 kcal mol^–1^, respectively. Nevertheless, both of these values are found in excellent agreement with the experimentally determined zero difference in the binding affinity following deuteration (Table 1). Nevertheless, we can conclude that the deuteration of these two systems does not exert any impact on their antagonistic features nor on their thermodynamic binding parameters. On the other hand, quite contrary, deuteration has a notable effect on the binding of both agonists **1**–**2**. For **1**, the interaction with the receptor becomes lower by 2.38 kcal mol^–1^, being in excellent agreement with the measured value of 2.08 kcal mol^–1^. Alternatively, deuteration increases the binding of **2** by –1.10 kcal mol^–1^, again matching the experimental value of –0.49 kcal mol^–1^, thus increasing its potency toward the H2 receptor by almost 10 times. This is a particularly significant observation given the fact that 4-methylhistamine **2** was originally described as human H2 agonist [53], but, regardless of the low sequence identity of the human H2 and H4 receptors (22%), **2** turned out to be a potent and selective full H4 receptor agonist with more than 100-fold selectivity for the H4 receptor over the other histamine receptor subtypes. Since **2** is well accessible, it has become the most frequently used human H4 receptor agonist [24]. Let us mention that our previous results position histamine in between these two agonists, with calculated and experimentally measured increase in the binding of –0.51 and –0.73 kcal mol^–1^, respectively [13]. The agreement between these sets of data is rather impressive, especially given the simplicity of the model employed for the quantization of nuclear motion performed on only a small but carefully selected part of the receptor molecule. However, it should be clear that the activation of GPCRs is a complex and dynamic process, linked with large conformational changes between receptor states. These are difficult to investigate experimentally, while, at the same time, occurring on time scales that are too slow for direct molecular simulations. However, it is gratifying to know that, for membrane receptors, high-resolution structures of the active and inactive conformation are slowly becoming available, which promotes understanding the nature of GPCR receptor activation on the atomic and electronic level as a foremost challenge [54]. Nevertheless, the results of this study offer convincing arguments that hydrogen bonding interactions are involved in the receptor activation and strongly demonstrate that deuteration can have a significant impact on the binding. This opens the door for the development of perdeuterated drugs, which could have different, in some instances more favorable clinical profile to already marketed substances. In finishing this section, we would like to emphasize that very recently, the US Food and Drug Administration approved the first deuterated drug, Austedo (deutetrabenazine), for the treatment of Huntington´s-disease-related movement disorders [55]. Austedo is also the first new treatment in over a decade for this indication, and preliminary studies show it is more efficacious [56] and is better tolerable [57] than its non-deuterated analogue.

## 4. Conclusions

This study reveals the importance of the hydrogen bonding interactions for the binding of histaminergic ligands to the histamine H2 receptor and computationally evaluates how these are affected by deuteration. We considered two agonists, 2-methylhistamine (**1**) and 4-methylhistamine (**2**), and two antagonists, cimetidine (**3**) and famotidine (**4**), and performed DFT calculations on a truncated model of the receptor´s binding site including Asp98, Asp186, Tyr250, Lys175, and Thr190 residues, in line with our previous studies. The overall binding was delineated in two contributions, that arising from the interaction with the receptor and the one originating from the interaction with the solvent preceding the binding. These were both modeled with the implicit CPCM solvation associated with the corresponding dielectric constants of ε = 4.0 for the receptor environment, and ε = 78.4 for the aqueous solution. The effect of the isotope substitution was introduced through an implicit quantization, by 2.3% shortening of all acidic N–H and O–H bonds [41,42,43,44,45,46].

The results show that both antagonists show weaker interactions with both the receptor and water solution than their agonist counterparts, and that these are only marginally affected by deuteration. As a result, our calculations predict practically no differences in the binding energies ΔΔ*E*_BIND,CALC_ for **3** and **4**, which is found in excellent agreement with experiments. In addition, our preliminary results for the antagonist mepyramine also show no effect of deuteration on its affinity for the H2 receptor (Figure S3). On the other hand, both agonists **1** and **2** are more polar and charged monocationic species, and their ability to interact with the solvent or the receptor binding site is much higher relative to uncharged antagonists considered here. Accordingly, deuteration exerts a much evident impact on these interactions, and our calculations show that **1** interacts less strongly with the H2 receptor by ΔΔ*E*_BIND,CALC_(**1**) = 2.38 kcal mol^–1^ upon deuteration, while the same effect works in the opposite direction for **2** increasing the overall binding by ΔΔ*E*_BIND,CALC_(**2**) = –1.10 kcal mol^–1^. Interestingly, these are also found in excellent match with experiments, which predict ΔΔ*E*_BIND,EXP_ of 2.08 kcal mol^–1^ for **1** and –0.49 kcal mol^–1^ for **2**. The obtained agreement between these sets of data is impressive, particularly given the simplicity of the model used here for the quantization of nuclear motion performed on only a small but carefully selected part of the receptor.

The results of this study provide convincing arguments that hydrogen bonding interactions are involved in the receptor activation, and strongly demonstrate that deuteration can have a significant impact on the binding. This opens the door for the development of perdeuterated drugs, which could have different, in some instances, more favorable, clinical profile to already marketed systems, and further progress in this area is highly recommended. In addition, the selective replacement of exchangeable hydrogen atoms with deuterium does not significantly impact the pharmacological profile of drugs and can elongate the duration of action due to slower decomposition [58]. Clinical trials of deuterated drugs are in progress [59], and there is still a long way towards the proper understanding of the receptor activation. Still, besides traditional methods of molecular pharmacology, computational work will play an important role in clarifying this process, which is likely to lead towards improved understanding of receptor activation and the design of new drugs.

## Figures and Tables

**Table 1:** Calculated deuteration-induced changes in the hydration energy (Δ*E*_HYDR_), H2 receptor interaction energy (Δ*E*_INTER_) and the overall receptor binding energy (Δ*E*_BIND_) as obtained by the (CPCM)/M06–2X/6–31+G(d,p) model. Experimental ΔΔ*E*_BIND,EXP_ values are taken from reference 13.

Ligand	In H_2_O			In D_2_O			ΔΔ*E*_BIND,CALC_	ΔΔ*E*_BIND,EXP_
	Δ*E*_HYDR_	Δ*E*_INTER_	Δ*E*_BIND_	Δ*E*_HYDR_	Δ*E*_INTER_	Δ*E*_BIND_		
2-methylhistamine (1)	–66.92	–71.99	–5.07	–66.88	–69.57	–2.69	2.38	2.08
4-methylhistamine (2)	–67.38	–86.13	–18.75	–67.55	–87.40	–19.85	–1.10	–0.49
cimetidine (3)	–19.69	–27.27	–7.58	–19.54	–27.21	–7.67	–0.09	0.00
famotidine (4)	–14.29	–31.17	–17.88	–13.97	–31.90	–17.93	–0.05	0.00

## Supplementary Materials

The following are available online at https://www.mdpi.com/2218-273X/10/2/196/s1, Figure S1: Computational scheme of **2** and **4** interacting with water molecules to calculate the energy of hydration; Figure S2: Computational scheme to calculate the interaction energy between **2** and **4** with the receptor binding site; Figure S3: Inhibition of the specific ^3^H-tiotidine binding to the histamine H2 receptor with mepyramine, revealing that deuteration did not cause any change in its affinity.

## References

[1] Deupi X., Kobilka B. (2007). Activation of G protein-coupled receptors. Adv. Protein Chem..

[2] Fredriksson R., Lagerstrom M.C., Lundin L.G., Schioth H.B. (2003). The G-protein-coupled receptors in the human genome form five main families. Phylogenetic analysis, paralogon groups, and fingerprints. Mol. Pharm..

[3] Cong X., Topin J., Golebiowski J. (2017). Class A GPCRs: Structure, function, modeling and structure-based ligand design. Curr. Pharm. Des..

[4] Congreve M., Langmead C.J., Mason J.S., Marshall F.H. (2011). Progress in structure based drug design for G-protein-coupled receptors. J. Med. Chem..

[5] Keshelava A., Solis G.P., Hersch M., Koval A., Kryuchkov M., Bergmann S., Katanaev V.L. (2018). High capacity in G protein-coupled receptor signaling. Nat. Commun..

[6] Franco M.I., Turin L., Mershin A., Skoulakis E.M.C. (2011). Molecular vibration-sensing component in drosophila melanogaster olfaction. Proc. Natl. Acad. Sci. USA.

[7] Turin L. (1996). A spectroscopic mechanism for primary olfactory reception. Chem. Senses.

[8] Turin L. (2002). A method for the calculation of odor character from molecular structure. J. Biol..

[9] Huelga S.F., Plenio M.B. (2013). Vibrations, quanta and biology. Contemp. Phys..

[10] Bittner E.R., Madalan A., Czader A., Roman G. (2012). Quantum origins of molecular recognition and olfaction in Drosophila. J. Chem. Phys..

[11] Hettinger T.P. (2011). Olfaction is a chemical sense, not a spectral sense. Proc. Natl. Acad. Sci. USA.

[12] Warshel A., Bora R.P. (2016). Defining and quantifying the role of dynamics in enzyme catalysis. J. Chem. Phys..

[13] Kržan M., Vianello R., Maršavelski A., Repič M., Zakšek M., Kotnik K., Fijan E., Mavri J. (2016). The quantum nature of drug-receptor interactions: Deuteration changes binding affinities for histamine receptor ligands. PLoS ONE.

[14] Ubbelohde A.R., Gallagher K.J. (1955). Acid-base effects in hydrogen bonds in crystals. Acta Cryst..

[15] Shao L., Hewitt M.C. (2010). The kinetic isotope effect in the search for deuterated drugs. Drug News Perspect..

[16] Guengerich F.P. (2013). Kinetic deuterium isotope effects in cytochrome P450 oxidation reactions. J. Label. Comp. Radiopharm..

[17] Shao L., Abolin C., Hewitt M.C., Koch P., Varney M. (2006). Derivatives of tramadol for increased duration of effect. Bioorg. Med. Chem. Lett..

[18] Russak E.M., Bednarczyk E.M. (2018). Impact of deuterium substitution on the pharmacokinetics of pharmaceuticals. Ann. Pharm..

[19] DeWitt S.H., Maryanoff B.E. (2018). Deuterated drug molecules: Focus on FDA-approved deutetrabenazine. Biochemistry.

[20] Liu J.F., Harbeson S.L., Brummel C.L., Tung R., Silverman R., Doller D. (2017). A decade of deuteration in medicinal chemistry. Ann. Rep. Med. Chem..

[21] Schmidt C. (2017). First deuterated drug approved. Nat. Biotechnol..

[22] Gant T.G. (2014). Using deuterium in drug discovery: Leaving the label in the drug. J. Med. Chem..

[23] Olsson M.H.M., Søndergaard C.R., Rostkowski M., Jensen J.H. (2011). PROPKA3: Consistent treatment of internal and surface residues in empirical p*K*_a_ predictions. J. Chem. Theory Comput..

[24] Stark H. (2013). Histamine H4 receptor. A novel drug target for immunoregulation and inflammation.

[25] Jantratid E., Prakongpan S., Dressman J.B., Amidon G.L., Junginger H.E., Midha K.K., Barends D.M. (2006). Biowaiver monographs for immediate release solid oral dosage forms: Cimetidine. J. Pharm. Sci..

[26] Islam M.S., Narurkar M.M. (1993). Solubility, stability and ionization behaviour of famotidine. J. Pharm. Pharm..

[27] Frisch M.J., Trucks G.W., Schlegel H.B., Scuseria G.E., Robb M.A., Cheeseman J.R., Scalmani G., Barone V., Petersson G.A., Nakatsuji H. (2016). Gaussian 16.

[28] Liao R.Z., Georgieva P., Yu J.G., Himo F. (2011). Mechanism of mycolic acid cyclopropane synthase: A theoretical study. Biochemistry.

[29] Vianello R., Repič M., Mavri J. (2012). How are biogenic amines metabolized by monoamine oxidases?. Eur. J. Org. Chem..

[30] Maršavelski A., Vianello R. (2017). What a difference a methyl group makes: The selectivity of monoamine oxidase B towards histamine and *N*-methylhistamine. Chem. Eur. J..

[31] Tandarić T., Vianello R. (2019). Computational insight into the mechanism of the irreversible inhibition of monoamine oxidase enzymes by the antiparkinsonian propargylamine inhibitors rasagiline and selegiline. ACS Chem. Neurosci..

[32] Himo F. (2017). Recent trends in quantum chemical modeling of enzymatic reactions. J. Am. Chem. Soc..

[33] Blomberg M.R.A., Borowski T., Himo F., Liao R.-Z., Siegbahn P.E.M. (2014). Quantum chemical studies of mechanisms for metalloenzymes. Chem. Rev..

[34] Quesne M.G., Borowski T., de Visser S.P. (2016). Quantum mechanics/molecular mechanics modeling of enzymatic processes: Caveats and breakthroughs. Chem. Eur. J..

[35] Sousa S.F., Ribeiro A.J.M., Neves R.P.P., Brás N.F., Cerqueira N.M.F.S.A., Fernandes P.A., Ramos M.J. (2017). Application of quantum mechanics/molecular mechanics methods in the study of enzymatic reaction mechanisms. Wires Comput. Mol. Sci..

[36] Quesne M.G., Silveri F., de Leeuw N.H., Catlow C.R.A. (2019). Advances in sustainable catalysis: A computational perspective. Front. Chem..

[37] Isom D.G., Castaneda C.A., Cannon B.R., Garcia-Moreno E.B. (2011). Large shifts in p*K*_a_ values of lysine residues buried inside a protein. Proc. Natl. Acad. Sci. USA.

[38] Harms M.J., Castaneda C.A., Schlessman J.L., Sue G.R., Garcia-Moreno E.B. (2009). The p*K*_a_ values of acidic and basic residues buried at the same internal location in a protein are governed by different factors. J. Mol. Biol..

[39] Hodošček M., Hadži D. (1989). Proton transfer in the HCOOH·CH_3_NH_2_ complex. Ab initio study with various basis sets and solvent reaction field. J. Mol. Struct..

[40] Scheiner S., Duan X. (1991). Effect of intermolecular orientation upon proton transfer within a polarizable medium. Biophys. J..

[41] De Souza J.M., Freire P.T.C., Bordallo H.N., Argyriou D.N. (2007). Structural isotopic effects in the smallest chiral amino acid: Observation of a structural phase transition in fully deuterated alanine. J. Phys. Chem. B.

[42] Shi C., Zhang X., Yu C.-H., Yao Y.-F., Zhang W. (2018). Geometric isotope effect of deuteration in a hydrogen-bonded host-guest crystal. Nat. Commun..

[43] Rivera-Rivera L.A., Wang Z., McElmurry B.A., Willaert F.F., Lucchese R.R., Bevan J.W., Suenram R.D., Lovas F.J. (2010). A ground state morphed intermolecular potential for the hydrogen bonded and van der Waals isomers in OC:HI and a prediction of an anomalous deuterium isotope effect. J. Chem. Phys..

[44] Mishra A.K., Murli C., Sharma S.M. (2008). High pressure Raman spectroscopic study of deuterated γ-glycine. J. Phys. Chem. B.

[45] Goncalves R.O., Freire P.T.C., Bordallo H.N., Lima J.A., Melo F.E.A., Mendes Filho J., Argyriou D.N., Lima R.J.C. (2009). High-pressure Raman spectra of deuterated L-alanine crystal. J. Raman Spectrosc..

[46] Smirnov S.N., Golubev N.S., Denisov G.S., Benedict H., Schah-Mohammedi P., Limbach H.-H. (1996). Hydrogen/deuterium isotope effects on the NMR chemical shifts and geometries of intermolecular low-barrier hydrogen-bonded complexes. J. Am. Chem. Soc..

[47] Vianello R., Mavri J. (2012). Microsolvation of the histamine monocation in aqueous solution: The effect on structure, hydrogen bonding ability and vibrational spectrum. New J. Chem..

[48] Wheeler S.E. (2012). Homodesmotic reactions for thermochemistry. Wiley Interdiscip. Rev. Comput. Mol. Sci..

[49] Perdan-Pirkmajer K., Mavri J., Kržan M. (2010). Histamine (re)uptake by astrocytes: An experimental and computational study. J. Mol. Model..

[50] Llinàs A., Glen R.C., Goodman J.M. (2008). Solubility challenge: Can you predict solubilities of 32 molecules using a database of 100 reliable measurements?. J. Chem. Inf. Model..

[51] Birdsall N.J. (1991). Cloning and structure-function of the H2 histamine receptor. Trends Pharm. Sci..

[52] Gantz I., DelValle J., Wang L.D., Tashiro T., Munzert G., Guo Y.J., Konda Y., Yamada T. (1992). Molecular basis for the interaction of histamine with the histamine H2 receptor. J. Biol. Chem..

[53] Black J.W., Duncan W.A., Durant C.J., Ganelin C.R., Parsons E.M. (1972). Definition and antagonism of histamine H2-receptors. Nature.

[54] Tehan B.G., Bortolato A., Blaney F.E., Weir M.P., Mason J.S. (2014). Unifying family A GPCR theories of activation. Pharm..

[55] Heo Y.A., Scott L.J. (2017). Deutetrabenazine; a review in chorea associated with Huntington’s disease. Drugs.

[56] Claassen D.O., Carroll B., De Boer L.M., Wu E., Ayyagari R., Gandhi S., Stamler D. (2017). Indirect tolerability comparison of Deutetrabenazine and Tetrabenazine for Huntington disease. J. Clin. Mov. Disord..

[57] Dean M., Sung V.W. (2018). Review of deutetrabenazine: A novel treatment for chorea associated with Huntington s disease. Drug Des. Devel. Ther..

[58] Kaur S., Gupta M. (2017). Deuteration as a tool for optimization of metabolic stability and toxicity of drugs. Glob. J. Pharmaceu. Sci..

[59] Tung R.D. (2016). Deuterium medicinal chemistry comes of age. Future Med. Chem..

