# Supplementary material for: Relevance of Hydrogen Bonds for the Histamine H2 Receptor-Ligand Interactions: A Lesson from Deuteration
Source: Biomolecules. 2020 Jan 29;10(2):196. doi: 10.3390/biom10020196 (PMC7072573; doi:10.3390/biom10020196)
Supplement: Supplementary file 1 [file biomolecules-10-00196-s001.pdf]

Supporting Information

Relevance of hydrogen bonds for the histamine H2 receptor-ligand interactions: A lesson from deuteration†

Mojca Kržan [1‡], Jan Keuschler [1‡], Janez Mavri [2], Robert Vianello [3,*]

[1] Institute of Pharmacology and Experimental Toxicology, Faculty of Medicine, University of Ljubljana, Vrazov trg 2, Ljubljana, Slovenia

[2] Laboratory for Computational Biochemistry and Drug Design, National Institute of Chemistry, Hajdrihova 19, Ljubljana, Slovenia

[3] Division of Organic Chemistry and Biochemistry, Ruđer Bošković Institute, Bijenička cesta 54, Zagreb, Croatia. Tel.: +385-1-4561117. Fax: +385-1-4680084. Email: robert.vianello@irb.hr

† This paper is dedicated to the memory of Prof. Dušan Hadži (1921–2019), a prominent researcher with a large impact on the hydrogen bond research, who recently passed away

‡ Both authors contributed equally to this work

**CONTENTS**	**PAGE**
**Figure S1.** Computational scheme of 4-methylhistamine monocation **2** (top) and famotidine **4** (bottom) interacting with water molecules to calculate the energy of hydration. The choice of the dielectric constant is indicated in round brackets.	S1
**Figure S2.** Computational scheme to calculate the interaction energy between 4-methylhistamine monocation **2** (top) and famotidine **4** (bottom) with the receptor binding site. The choice of the dielectric constant is indicated in round brackets.	S2
**Figure S3.** Inhibition of the specific ^{3}H-tiotidine binding to the histamine H2 receptor with mepyramine. The obtained IC_{50} values are 7.6 ± 0.17 (in H_2O) and 7.6 ± 2.2 (in D_2O), which reveal that deuteration did not cause any change in the affinity of this antagonist.	S3

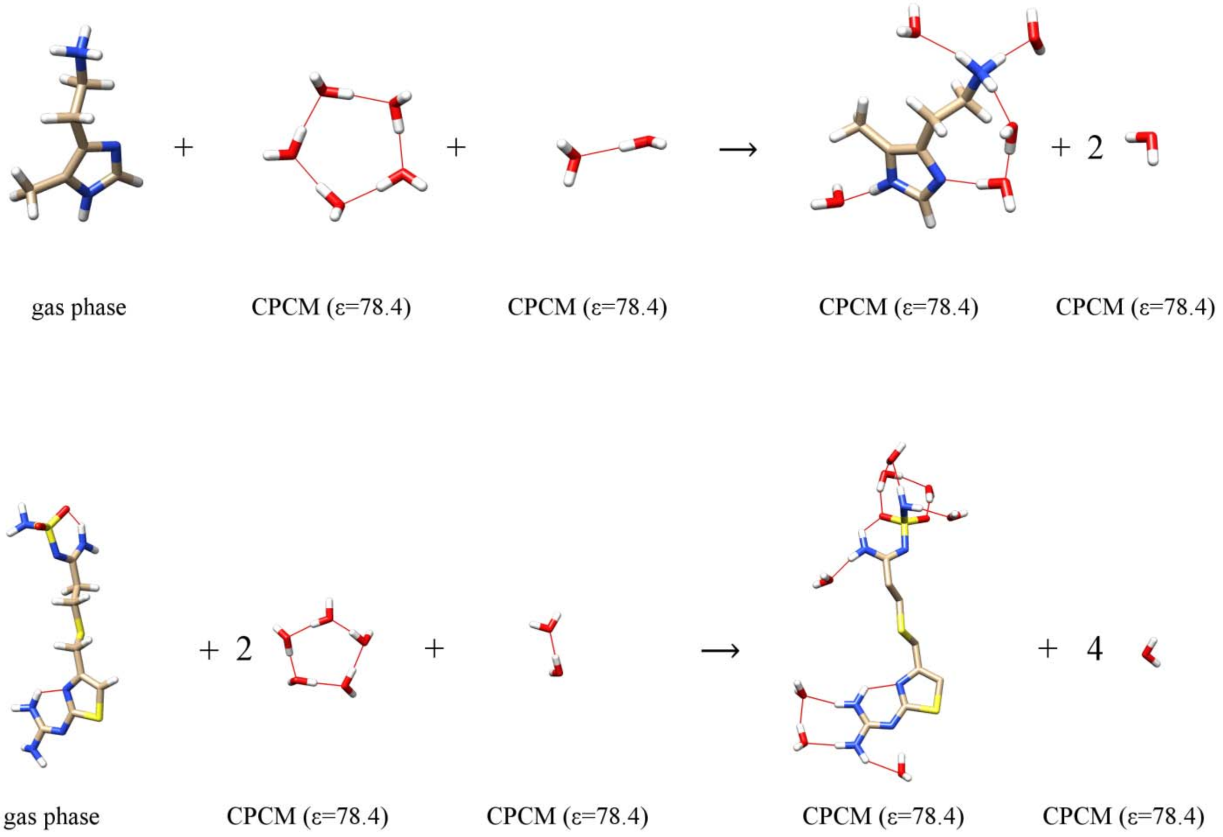

Figure S1. Computational scheme of 4-methylhistamine monocation **2** (top) and famotidine **4** (bottom) interacting with water molecules to calculate the energy of hydration. The choice of the dielectric constant is indicated in round brackets.

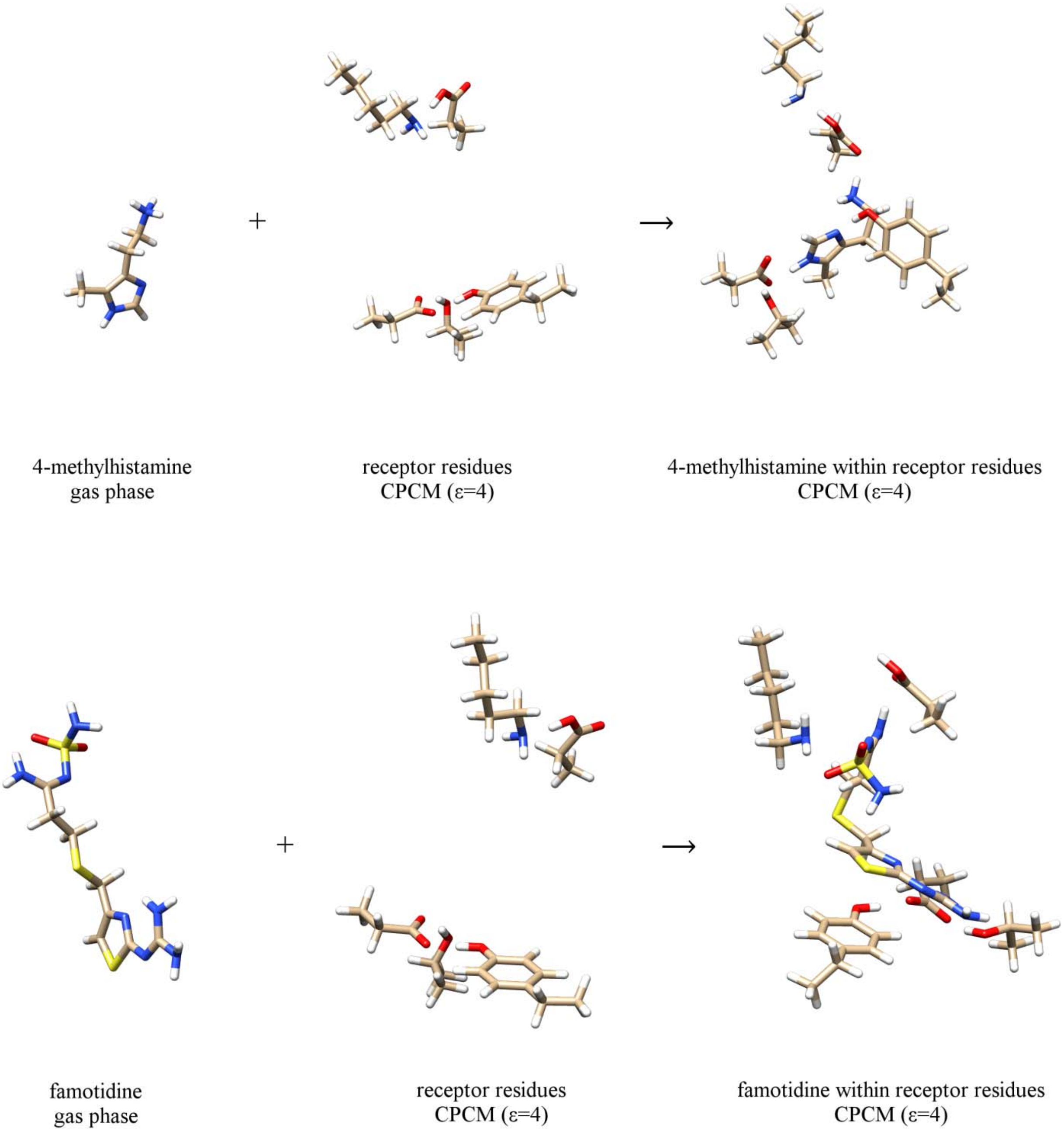

Figure S2. Computational scheme to calculate the interaction energy between 4-methylhistamine monocation **2** (top) and famotidine **4** (bottom) with the receptor binding site. The choice of the dielectric constant is indicated in round brackets.

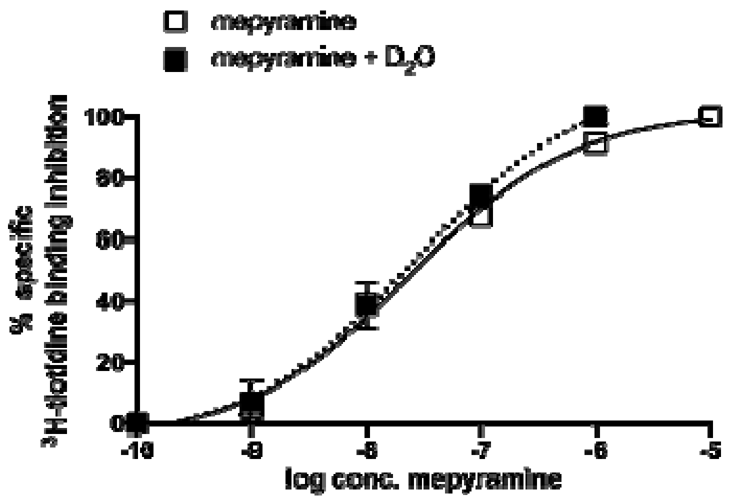

Figure S3. Inhibition of the specific ^{3}H-tiotidine binding to the histamine H2 receptor with mepyramine. The obtained IC_{50} values are 7.6 ± 0.17 (in H_2O) and 7.6 ± 2.2 (in D_2O), which reveal that deuteration did not cause any change in the affinity of this antagonist.
